# Correction to ‘Introduced bees (*Osmia cornifrons*) collect pollen from both coevolved and novel host-plant species within their family-level phylogenetic preferences’

**DOI:** 10.1098/rsos.201375

**Published:** 2020-08-26

**Authors:** Anthony D. Vaudo, David J. Biddinger, Wiebke Sickel, Alexander Keller, Margarita M. López-Uribe

*R. Soc. Open Sci.* **7**, 200225 (published Online 22 July 2020) (doi:10.1098/rsos.200225)

This correction concerns figure 1. The plant genus on the x-axis was given as ‘Eubus’ instead of ‘Rubus’. This has now been corrected.[Fig RSOS201375F1]

**Figure 1. RSOS201375F1:**
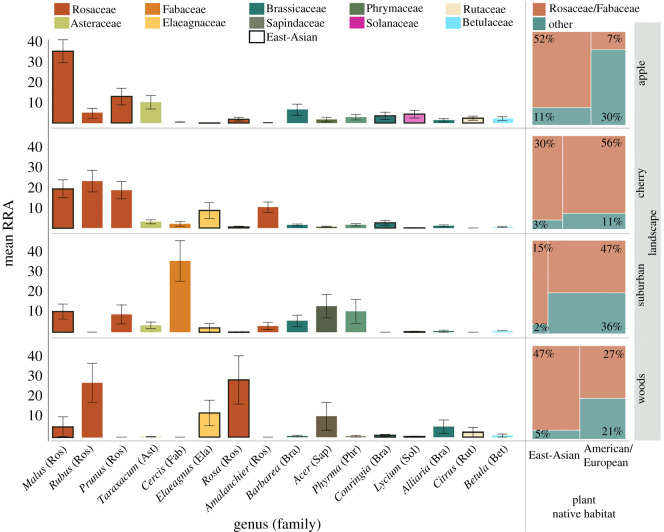
*Osmia cornifrons* larval provision host-plant genera by landscape coloured by plant family. Data in left panel are mean relative read abundances ± s.e. Boxes outlined in black are East-Asian origin. Right panel indicates proportions of pollen collected from East-Asian and/or Rosaceae/Fabaceae pollen versus not. Note that Rosaceae genera are the most represented across all landscapes and East-Asian abundances vary by landscape.

