# Supplementary figures and images for: Introduced bees (Osmia cornifrons) collect pollen from both coevolved and novel host-plant species within their family-level phylogenetic preferences
Source: R Soc Open Sci. 2020 Jul 22;7(7):200225. doi: 10.1098/rsos.200225 (PMC7428236; doi:10.1098/rsos.200225)

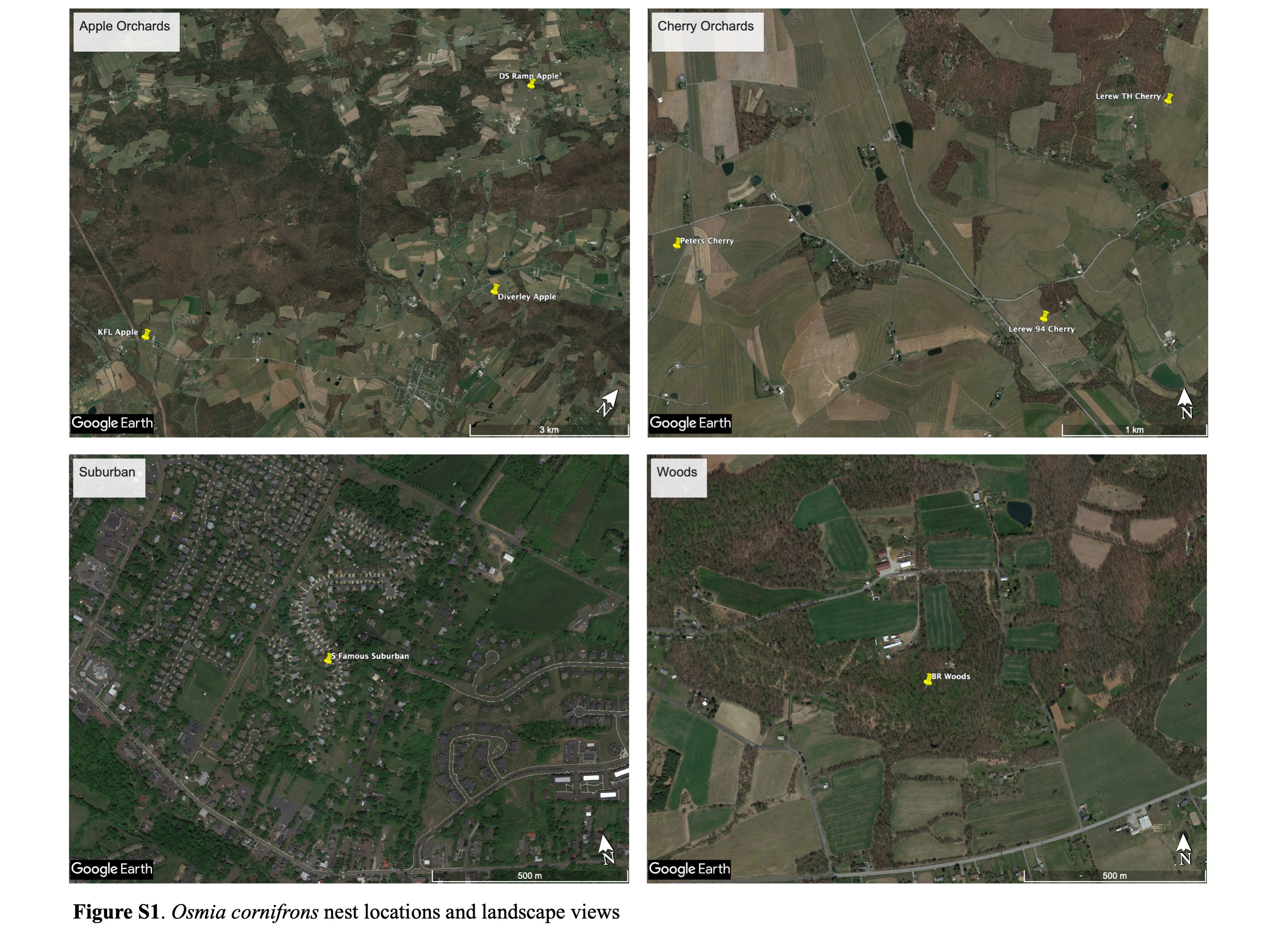

Supplement: Figure S1 [file rsos200225supp1.jpeg]

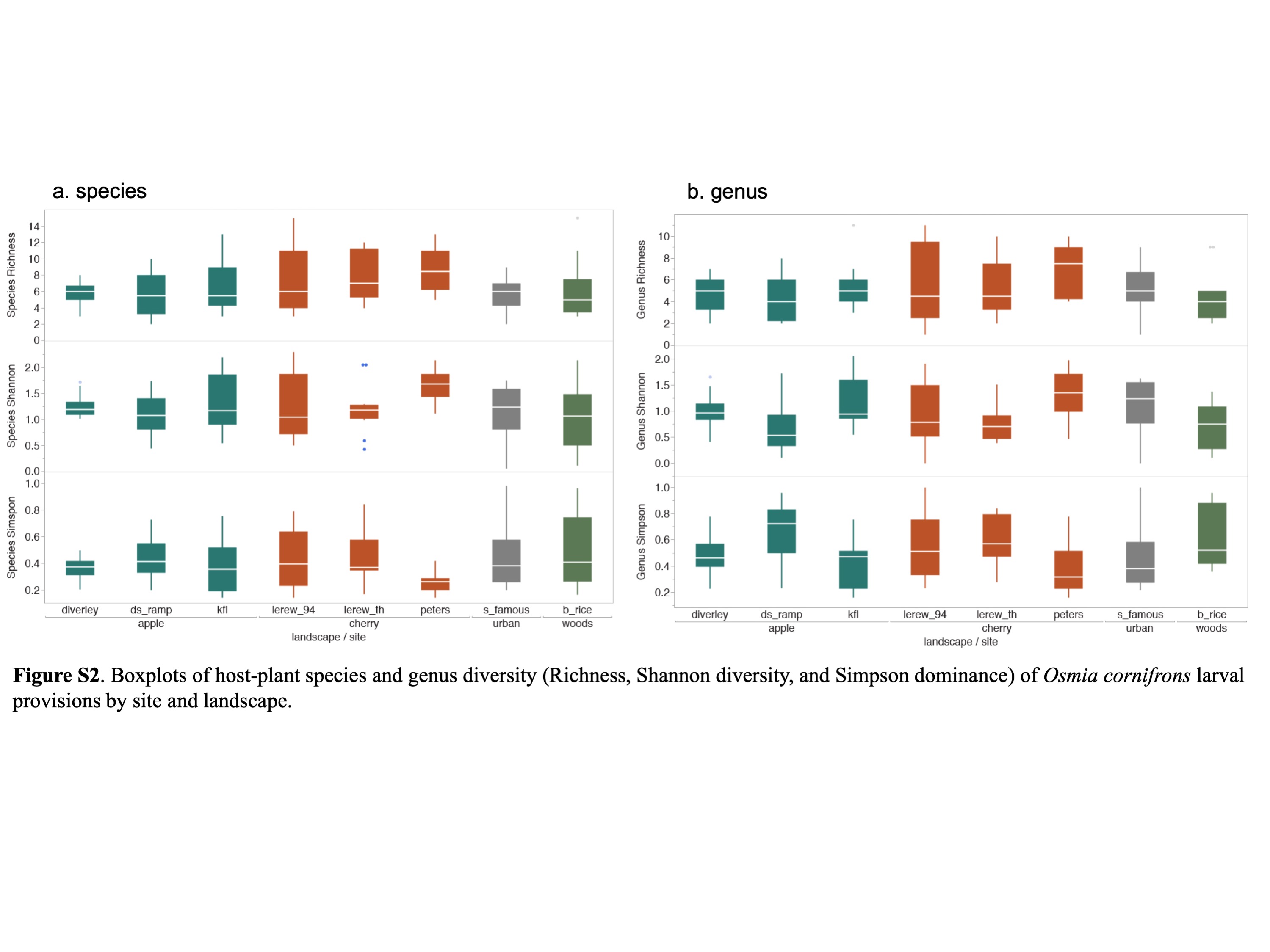

Supplement: Figure S2 [file rsos200225supp2.jpg]

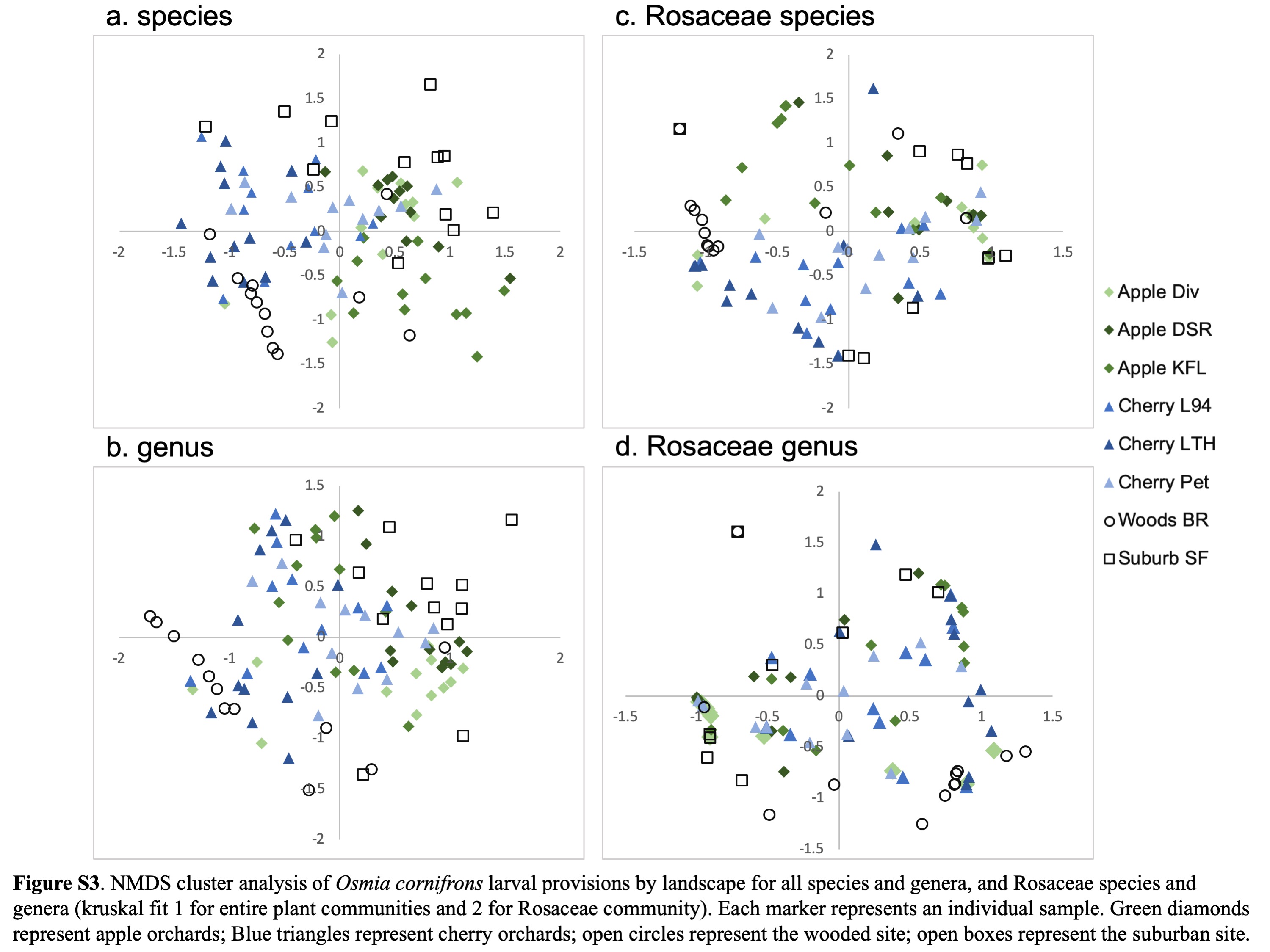

Supplement: Figure S3 [file rsos200225supp3.jpg]

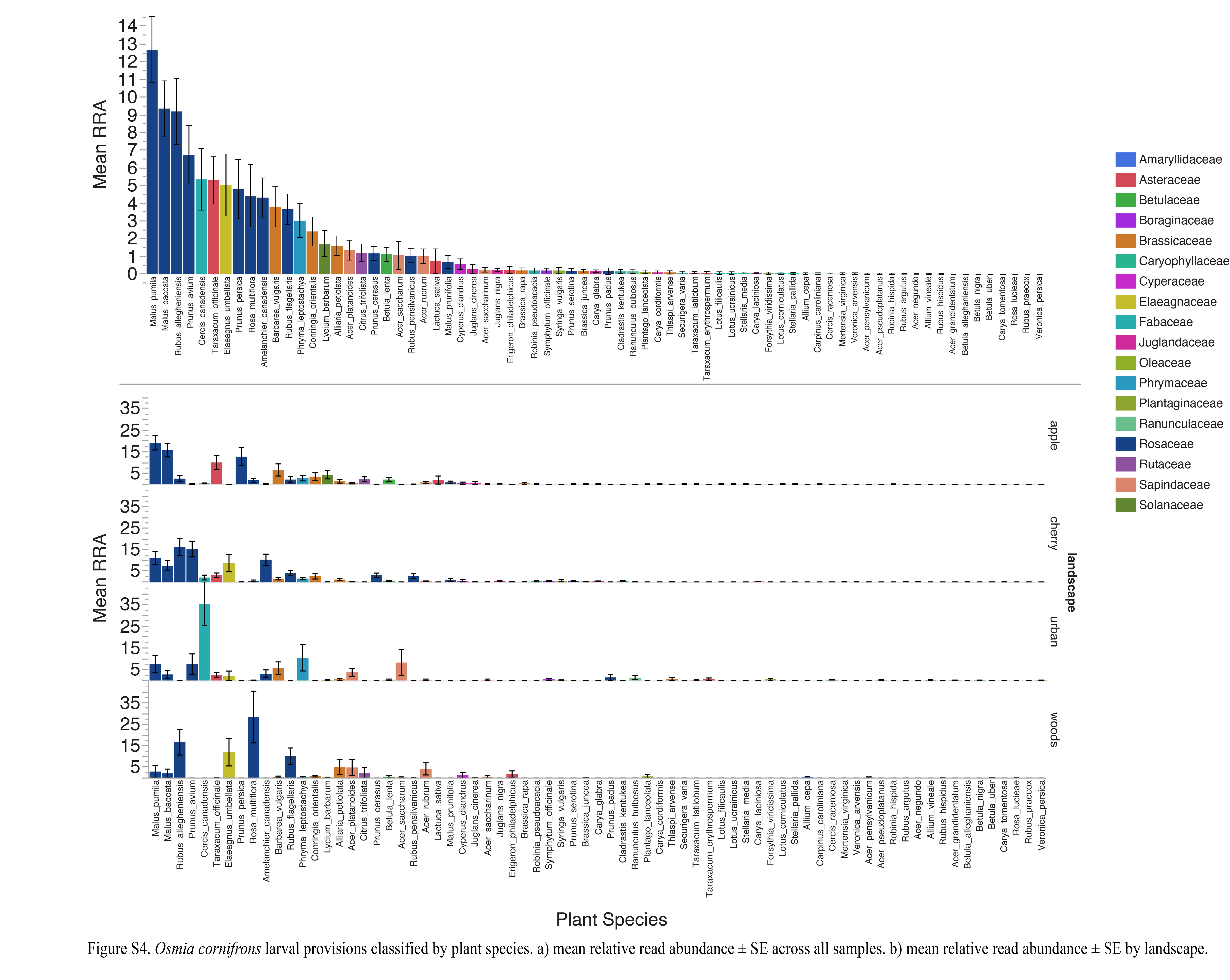

Supplement: Figure S4 [file rsos200225supp4.jpg]

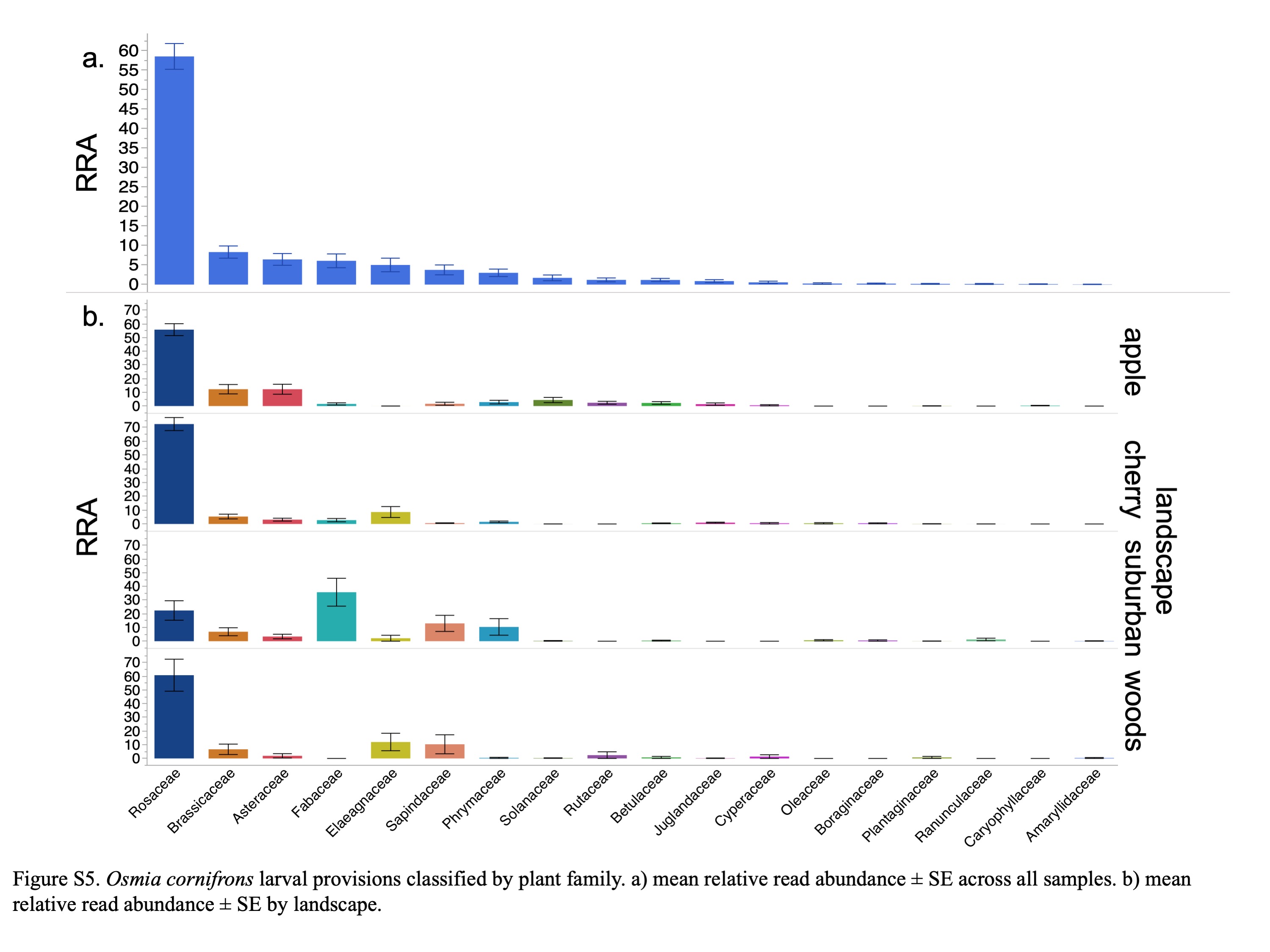

Supplement: Figure S5 [file rsos200225supp5.jpeg]
